# Complete mitochondrial genome of the Endangered species *Ellobium chinense* (Pulmonata, Ellobiidae) from Korea

**DOI:** 10.1080/23802359.2016.1261609

**Published:** 2017-01-04

**Authors:** Jumin Jun, Eun Hwa Choi, Hyun Jong Kil

**Affiliations:** a Animal Resources Division, National Institute of Biological Resources, Seo-gu Incheon, Republic of Korea;; b Division of EcoScience, Ewha Womans University, Seoul, Republic of Korea;; c Institute for Phylogenomics and Evolution, Kyungpook National University, Daegu, Republic of Korea

**Keywords:** Pulmonata, Ellobiidae, Endangered species, mitochondrial genome, *Ellobium chinense*

## Abstract

The complete mitochondrial genome of *Ellobium chinense* (Ellobioidea, Ellobiidae), an Endangered species in South Korea, is reported here for the first time. The mitogenome of *E. chinense* is 13,979 base pairs in total length and includes 13 PCGs, small and large rRNAs, and 21 tRNAs. Twelve genes are encoded on the light-strand and 24 genes on the heavy-strand. Compared to four other ellobiid species, the PCGs of *E. chinense* have a conserved gene order except for the positions of *ND4L* and *ND4*. These data provide useful molecular information for phylogenetic studies concerning ellobiids and related species.

*Ellobium chinense,* a species of terrestrial pulmonate gastropod, is designated as an Endangered species in South Korea and is found mainly in the upper intertidal zone (The National Institute of Biological Resources 2014). Data are lacking on its biological characteristics and genetic information. In this study, we determined the complete mitochondrial genome of *E. chinense* (13,979 bp; GenBank accession number KY056647).

A specimen of *E*. *chinense* was collected from the upper intertidal zone in Gwangyang-si, Jellanam-do, South Korea. Genomic DNA was extracted (deposited in National Institute of Biological Resources, accession 120913G2) using a QIAmp DNeasy Blood & Tissue Kit (QIAGEN, Hilden, Germany). The mitochondrial genome sequence was obtained using next-generation sequencing, with raw reads generated from an Illumina Hi-seq 2500 (Illumina Inc., San Diego, CA). Using CLC genomic workbench 9.0.1 (CLC Bio-Qiagen, Aarhus, Denmark), 6,282 out of 12,038,798 reads were assembled to produce a circular form of the complete mitochondrial genome with an average of 67 × coverage.

The mitochondrial genome contains large and small rRNAs, 21 tRNAs, and 13 PCGs. tRNAs and PCGs were predicted using tRNAscan-SE 1.21 (Schattner et al. 2005), ARWEN (Laslett & Canbäck 2008), and ORFfinder (https://www.ncbi.nlm.nih.gov/orffinder/). The overall base composition of the mtDNA is 24.4% A, 18.6% C, 33.9% T, and 23.1% G. Most of the genes are encoded on the heavy strand, except for four PCGs (*ATP6*, *ATP8*, *ND3*, and *COIII*) and seven tRNA genes (*tRNA*^Gln^, *tRNA*^Leu^, *tRNA*^Asn^, *tRNA*^Met^, *tRNA*^Thr^, *tRNA*^Glu^, and *tRNA*^Arg^).

Five protein-coding genes (*ND1, ND4, ND4L, ATP8*, and *COIII*) are initiated by the ATG start codon, and six PCGs (*COI, COII, ND5, ATP6, CYTB*, and *ND3*) are initiated by TTG. The *ND6* and *ND2* genes have ATT and GTG as the start codon, respectively. Four genes (*ND2*, *ND3*, *ND5*, and *COI*) end on the canonical stop codon TAG and another four genes (*ND1*, *ND4*, *ND6*, and *COII*) use TAA as the stop codon. The remaining five genes (*ND4L*, *CYTB*, *ATP6*, *ATP8*, and *COIII*) have an incomplete stop codon and have a terminal T or TA. The tRNAs are spread over the whole genome and their lengths vary from 60 bp to 79 bp. While four other species (*Auriculinella bidentata, Ovatella vulcani, Myosotella myositis*, and *Pedis pedis*) in the same family have two *tRNA^ser^* genes, only one *tRNA^ser^* has been found on the mitochondrial genome of *E. chinense* (White et al. 2011). The gene order on the *E. chinense* mitochondrial genome is the same as previously described for two other ellobiid species, *A. bidentata* and *O. vulcani,* except for the *tRNA^ser^* number. In addition, the PCG arrangement of *E. chinense* is identical to the mitochondrial genome of *M. myosotis,* except for the position of *ND4L*, and is identical to *P. pedis*, except for the position of *ND4* (White et al. 2011).

The complete mitochondrial genomes of *E. chinense* and four other species from the same family were used to reconstruct a maximum-likelihood tree with 500 bootstrap replicates (Figure 1). The phylogenetic analyses were performed using MEGA 6.0 (Tamura et al. 2013).

**Figure 1.**
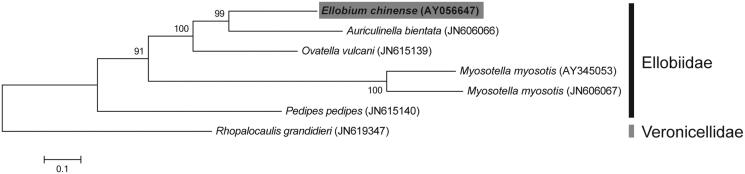
The molecular phylogenetic tree of *E. chinense* and other related species in Ellobiidae based on mitochondrial 13 protein-coding gene sequences. The complete mitochondrial genomes were obtained from GenBank and the accession numbers of the sequences are indicated in parentheses after scientific name of each species. The phylogenetic tree constructed by a maximum-likelihood method with 500 bootstrap replicates.
